# Medical Institution of Yale College

**Published:** 1845-02

**Authors:** 


### RECORD OF MEDICAL SCIENCE.

*Medical Institution of Yale College.*—The annual examination of Candidates in this Institution commenced on Wednesday, the 15th inst. and continued two days. Present on the part of the Connecticut Medical Society, Luther Ticknor, M. D. of Salisbury, President, Charles Woodward, M. D. of Middletown, Silas Fuller, M. D. of Hartford, Archibald Welch, M. D. of Wethersfield, and Rufus Blakeman, M. D. of Fairfield; and on the part of Yale College, Professors Silliman, Ives, Knight, Beers, Hooker and Bronson.

Eleven candidates, who had attended two full courses of lectures, and complied with the other legal requirements, were admitted to the degree of Doctor in Medicine, by President DAY, of Yale College, viz:

James Austin, B. A., Union College, Carmel, N. Y.—On "Remittent Fever."

Gardner Barlow, Meriden—On "The Demerits of Empiricism."

Edward McEwen Beardsley, Monroe—On "Puerperal Fever."

Joseph Edgar Clark, Rootstown, Ohio—On "Medical Chemistry."

Robert Wasson Forbes, B. A., New Haven—The Valedictory Address.

Benjamin Maltby Fowler, New Haven—On "The importance of the general diffusion of Elementary Medical Education."

Hiram Holt Loomis, Woodstock—On "The Fœtal Circulation."

William Henry Rossell, Trenton, N. J.—On "Ophthalmia."

John Hanson Thompson, Philadelphia, Pa.—On "Necrosis."

Edward Goodrich Ufford, South Hadley, Mass.—On "Scarlatina."

Enoch Tenney Winter, New Haven—On "Scrofula."

On Wednesday afternoon, the *Valedictory Address* was pronounced in the College Chapel by ROBERT WASSON FORBES, B. A., one of the candidates, and the *Annual Address* to the candidates by CHARLES WOODWARD, M. D. of Middletown, of the Board of Examiners.

WILLIAM H. COGSWELL, M. D. of Plainfield, is appointed to give the Annual Address to the candidates at the examination in 1846, and RUFUS BLAKEMAN, M. D. of Fairfield, his substitute.—*New Haven Daily Herald, Jan.* 18*th*, 1845.

